# Seasonal Patterns of Oestrus and Reproduction in Street Dogs of Indian Cities

**DOI:** 10.3389/fvets.2022.821424

**Published:** 2022-06-17

**Authors:** George Brill, Tamara Kartal, Dev Prakash Yadav, Mukesh Bhyan, Chirag Patel, Shiv Kumar Sheoran, Piyush Patel, Bimmy Painuly, Amit Chaudhari

**Affiliations:** Humane Society International, Washington, DC, United States

**Keywords:** street dog, sterilization, oestrus, pregnancy, pups

## Abstract

Understanding seasonal breeding dynamics is essential for maximizing efficiency and welfare in the application of street dog management programs. Humane Society International (HSI) has conducted many animal birth control programmes concerning the street dog populations in urban India. This paper analyses the data on reproductive indicators—oestrus, pregnancy, and pups—collected by HSI sterilization clinics in the cities of Jamshedpur, Dehradun, and Vadodara over a period of 3, 5, and 4 years, respectively. We found a consistent reproductive seasonality dynamic in all three cities, with peak oestrus and pregnancies occurring in the late/post-monsoon season. Pup proportions peaked soon after. Both these findings are consistent with previous studies of free-roaming domestic dog populations both in India and worldwide. Additionally, we identified minor inter-city differences in the temporal breeding dynamic, which we propose are the result of localized seasonal climatic and human factors. Finally, we examine and assert the relevance of breeding seasonality in the implementation of efficient and welfare-sensitive birth control programmes.

## Introduction

Since 2012, Humane Society International (HSI) has assessed and monitored street dog populations in cities and rural settlements worldwide, including countries in South and Southeast Asia, Latin America, and Africa. In many of these regions, HSI managed birth control and welfare programmes have been established. In particular, a widespread sterilization drive is currently in operation across India, with HSI sterilization clinics having been run, or currently running, in more than ten Indian cities, having sterilized over 100,000 dogs since 2013.

A recurrent dynamic revealed in HSI's street dog monitoring surveys is the seasonal fluctuation of street dog abundance when examined on a biannual survey cycle (1)—the result of apparent breeding seasonality in the street dog population. Elsewhere, seasonality of oestrus has been observed amongst free-roaming dog populations in the Indian settlements of Jodhpur (2), Jaipur (3), West Bengal (4), Katwa town (5), Kolkata (6), Goa and Tamil Nadu (7); however, other studies have asserted that this dynamic is not universal to free-roaming populations of the species (8), nor for many confined populations (9, 10). Indeed, the reproductive cycle of the domestic dog (*Canis familiaris*) has long been considered non-seasonal (11, 12). For an extensive summary of published analyses of free-roaming and confined dog breeding dynamics see (7).

Understanding the seasonal breeding dynamic of a free-ranging animal population is essential for the effective design of animal birth-control (ABC) programmes, ensuring that they are implemented to maximize both programme efficiency and the welfare of the population they target. Additionally, awareness of a seasonal breeding dynamic is imperative for the implementation of accurate street dog monitoring programmes, especially with regards to the frequency and timing of follow-up surveys for the purposes of evaluating ABC programme success.

While biannual monitoring surveys may identify breeding seasonality, they do not possess the temporal resolution to describe precise oestrus dynamics, nor the capacity to examine the finer nature of a population's breeding cycle. Furthermore, broader fluctuations in population abundance due to the effects of sterilization, methodological challenges and other factors mean that recurrent measures of dog abundance are not an accurate representation of population fluctuation with regard to breeding seasonality specifically. In order to further understand such dynamics, it is necessary to ascertain the relative proportions of reproductive indicators within the population on a monthly basis in order to reveal patterns of seasonal fluctuation—a methodology utilized previously by a number of researchers [e.g., (2, 3, 7, 8)].

During ABC programmes conducted in India, HSI sterilization clinics have recorded data (13) on each animal operated on. In particular, the monthly proportion of reproductive indicators—pups, pregnant bitches, and bitches in oestrus—can provide insight into the breeding dynamics of each city's street dog population.

Many of HSI's ABC programmes have been implemented on a short-term or intermittent basis, the product of specific short-term sterilization drives, government-driven efforts, and inevitable inconsistency in funding availability. However, three cities—Jamshedpur, Dehradun, and Vadodara—possess long-running programmes of sufficient duration and consistency to examine the annual reproductive cycles of their respective street dog populations.

Expanding on similar studies conducted in other cities in the past [e.g., (2, 3, 7)] this analysis of sterilization clinic data is an attempt to comprehensively characterize breeding seasonality in three further Indian cities—Jamshedpur, Dehradun, and Vadodara—as well as provide an indication as to the predictability of seasonal dynamics of three interrelated reproductive indicators: oestrus, pregnancy and pup prevalence.

## Materials and Methods

### Study Area Locations

Street dog data were collected by the HSI sterilization clinic in each of three Indian cities: Jamshedpur (state of Jharkhand), Dehradun (state of Uttarakhand), and Vadodara (state of Gujarat). Jamshedpur (latitude: 22.805618, northeastern India) has a tropical climate, with average temperatures ranging from 12–39°C; the rainy season stretches from June to mid-October. Vadodara (latitude: 22.310696, western India) is also tropical, with a rainy season from mid-June to September, and temperature averages of 14–40°C. Dehradun (latitude: 30.316496, northern India) is subtropical with an average temperature range of 8–39°C, and monsoons occurring between July and September. Dehradun stands at an elevation of 640 m above sea level and experiences distinct summer-winter seasonality.

### Animal Population

**Figure 1:**
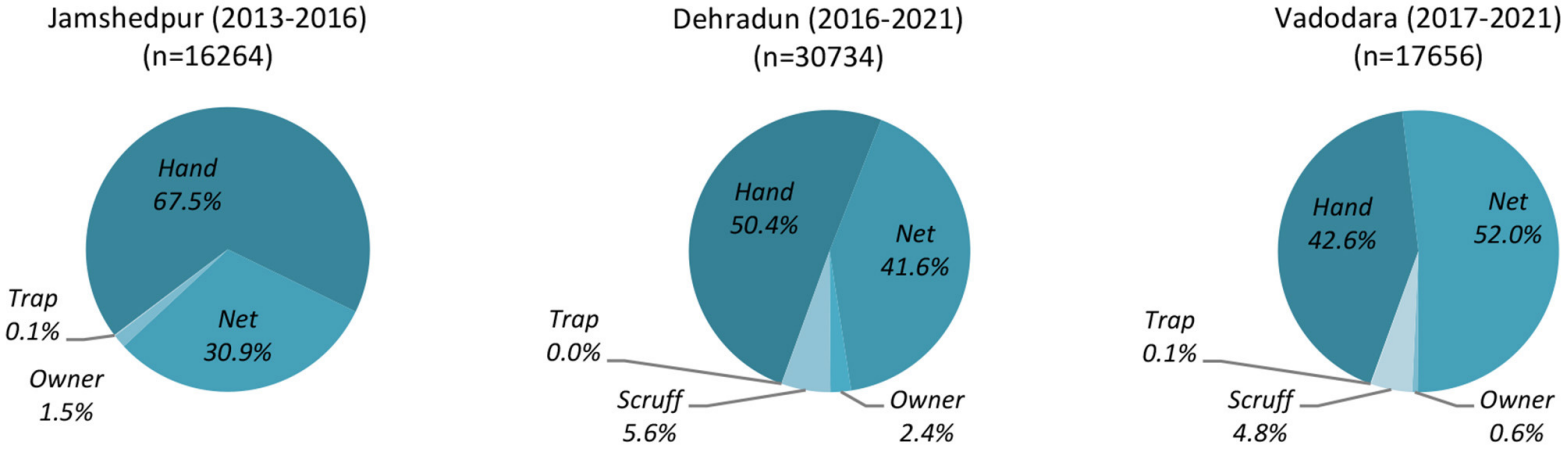
Clinic catch method in the Indian cities Jamshedpur, Dehradun, and Vadodara over the durations of data collection periods.

The dogs recorded in this study were free-roaming street dogs caught for the purposes of sterilization. Dogs were caught by mobile catch teams operating in collaboration with each city's HSI sterilization clinic. The catch methods employed by these teams in each city are detailed in Figure 1. A small number of additional dogs were brought in by “owners” [Jamshedpur: 251 (1.5%); Dehradun: 724 (2.4%); Vadodara: 125 (0.7%)] and may therefore be considered privately owned dogs. Subsequent to sterilization, all free-roaming dogs were re-released onto the streets. Since the data analyzed were drawn from already existing datasets, and was not collected for the purposes of research but rather comprise the operation records of veterinary clinics [operating under local and national guidelines—see especially (14)], ethical approval for this study was not required.

The catching and sterilization of pregnant females was avoided as per AWBI guidelines, and if identified after catching, pregnant females were returned to point of origin the following day. However, in many cases—typically of early- to mid-term—it was only possible to identify pregnancies after sedation or incision, at which point abortion and sterilization are considered to be the safest course of action due to the risk of fetal complication and death. HSI clinics follow the highest standards of veterinary procedures and unpublished data indicates that HSI's post-operative complication rate for pregnant females is lower even than that of other female dogs.

### Data Collection

Data were collected on each dog that entered the HSI sterilization clinics, including age, sex, and, in the case of adult bitches, the presence of oestrus or pregnancy (note that lactation status and pup dependencies of females were not recorded since lactating females were avoided according to HSI's sterilization protocol). Since the HSI clinics were set-up for the purposes of sterilization, only a very small number of individuals were brought in for other purposes, and are not included in the analyses, unless they too were sterilized as part of their treatment. Male or female dogs <7–8 months of age were considered pups; age was determined based on body weight (8–9 kg was considered pup); pups <5 kg (~4 months) were not caught. In the case of sterilization of pregnant adult bitches, the number of aborted fetuses removed simultaneously were recorded. For the purposes of this analysis, bitches were considered pregnant when a fetuses count was recorded. Adult bitches were checked for oestrus by a qualified veterinarian based upon inspection of the vulva for swelling or discharge. Dogs that were brought into the HSI clinics but were not operated on [Jamshedpur: 700 (4.3%); Dehradun: 766 (2.5%); Vadodara: 1,028 (4.9%)] were excluded from the data analysis. Of the dogs operated on, 15 and 7 individuals of unrecorded age and sex were excluded from the Jamshedpur and Dehradun analysis, respectively. Note that the street dog population of these cities largely consist of a single breed—the Indian pariah dog.

It is notable that city-specific guidelines and cultural beliefs will have impacted data collection in a number of ways. In Dehradun, as a result of a fatal anesthesia incident involving pups (believed to be related to the use of specific anesthetics at altitude), the catching of pups for sterilization was abandoned soon after the beginning of the programme. As a result, pup data is poorly recorded for Dehradun, and thus were not tested for seasonality (see Figure 5). Furthermore, in both Dehradun and Vadodara the catching of pregnant females was avoided, following local government guidelines. In practice, however, since only advanced pregnancies tend to be visually detectable, many early and mid-term pregnancies were caught and sterilized, thus providing a continuous proportion of pregnant bitches from which seasonality may be tested. The restriction on catching pregnant females is not believed to have a confounding effect on the results of the study since it remained in place to the same extent throughout the entire data collection period, and in both cities the number of pregnant females still represented significant proportions of the population. While selective catching may have acted to reduce the apparent magnitude of the seasonal pregnancy peak, it should not have impacted the temporal location of that increase. The same dynamic is true for the avoidance of pups under 5 kg. In Jamshedpur there were no restrictions on catching demographics, although it is of course likely that heavily pregnant and lactating females, in addition to very young pups, would typically have been hidden and thus less likely to have been captured in all three cities. While these catch biases are likely to have a significant effect on absolute proportions of the specific demographic categories caught, they similarly should not skew the temporal (seasonal) pattern within each demographic.

Data were collected through direct input into an online data recording system via the smartphone application SETU (13) by the veterinary team conducting the sterilization procedure. The data recording periods, and total number of dogs sterilized by each city's clinic are detailed in Table 1, noting a gap in data collection during April 2020 in Dehradun and Vadodara, and May 2021 in Dehradun as a result of circumstances pertaining to the Covid-19 pandemic.

**Table 1:** Data collection periods and total clinic sterilization counts in the Indian cities Jamshedpur, Dehradun, and Vadodara. Fifteen and seven dogs of unrecorded sex and age were excluded from the Jamshedpur and Dehradun datasets, respectively.

**City**	**Data collection period**	**No data collection**	**Total dogs sterilized**	**Total bitches sterilized** **(exc. pups)**
Jamshedpur	Jul 2013–Mar 2016	-	15,610	5,750
Dehradun	Nov 2016–Jul 2021	April 2020, May 2021 (Covid-19 pandemic)	29,982	15,565
Vadodara	Sep 2017–Jul 2021	April 2020 (Covid-19 pandemic)	19,978	8,860

### Statistical Methods

Data were analyzed on a city-by-city basis, with count data concerning the presence and absence of each reproductive indicator combined across years to give monthly totals, providing a more reliable representation of seasonal dynamics than individual year analyses. To test for seasonality of reproductive indicators (oestrus, pregnancy, and pups), a chi-squared test was conducted to determine if there was association between the prevalence of each indicator and months of the year. A chi-squared *post-hoc* test with *p*-value correction (Bonferroni) was then conducted to confirm the temporal continuity and unimodality of any seasonal variation (15); the results of these *post-hoc* tests are reported in Appendix A. All data analysis was performed in R (16).

## Results

### Jamshedpur

**Figure 2:**
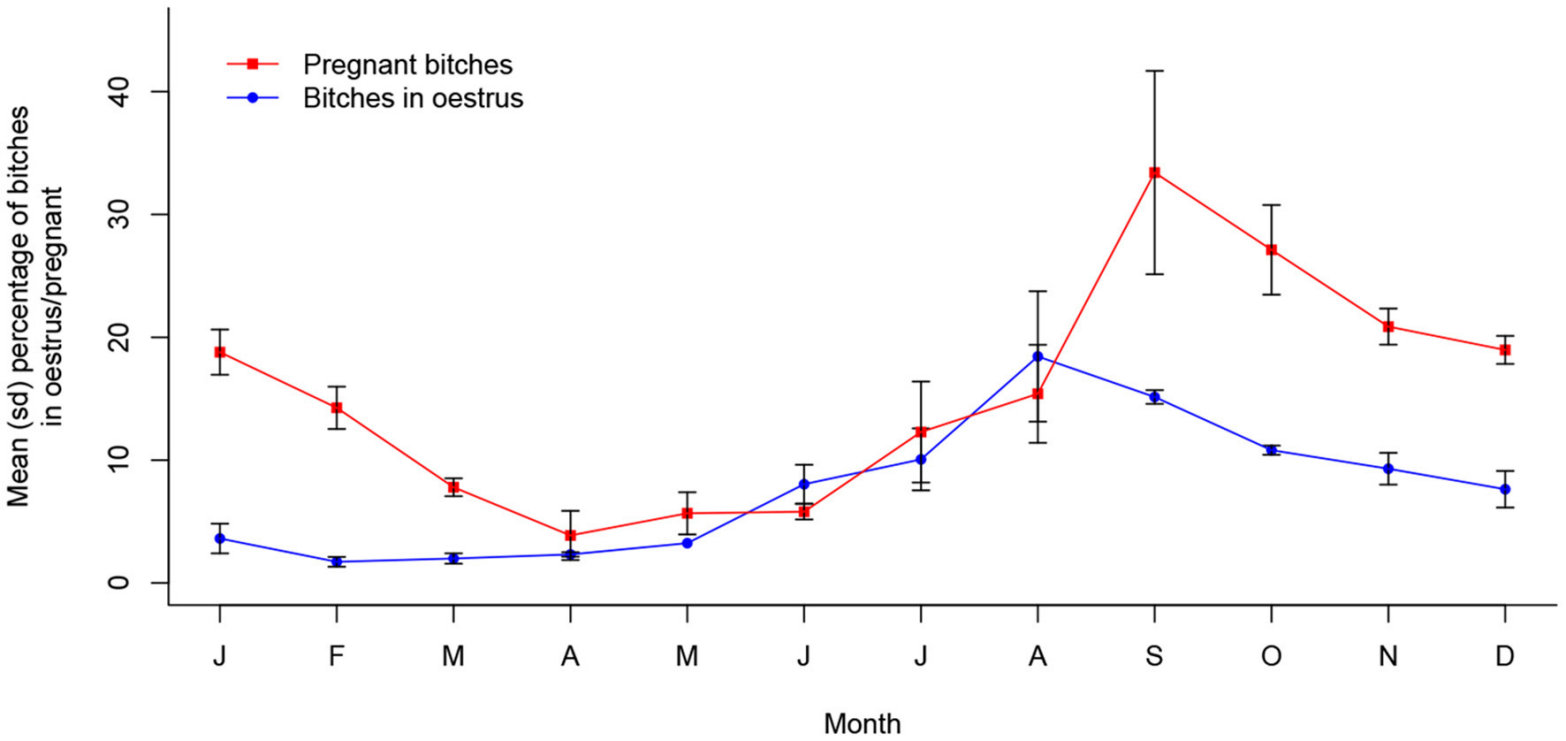
Mean (sd) proportions of bitches in oestrus and pregnant in Jamshedpur, India between 2013 and 2016 (*n* = 5,750).

In Jamshedpur, the prevalence of both oestrus (*X*^2^ (11, *N* = 5,750) = 221.72, *p* < 0.001) and pregnancy (*X*^2^ (11, *N* = 5,750) = 294.07, *p* < 0.001) was significantly associated with month of the year, with the annual peak period centered around August to September and September to November, respectively. This dynamic is shown in Figure 2.

**Figure 3:**
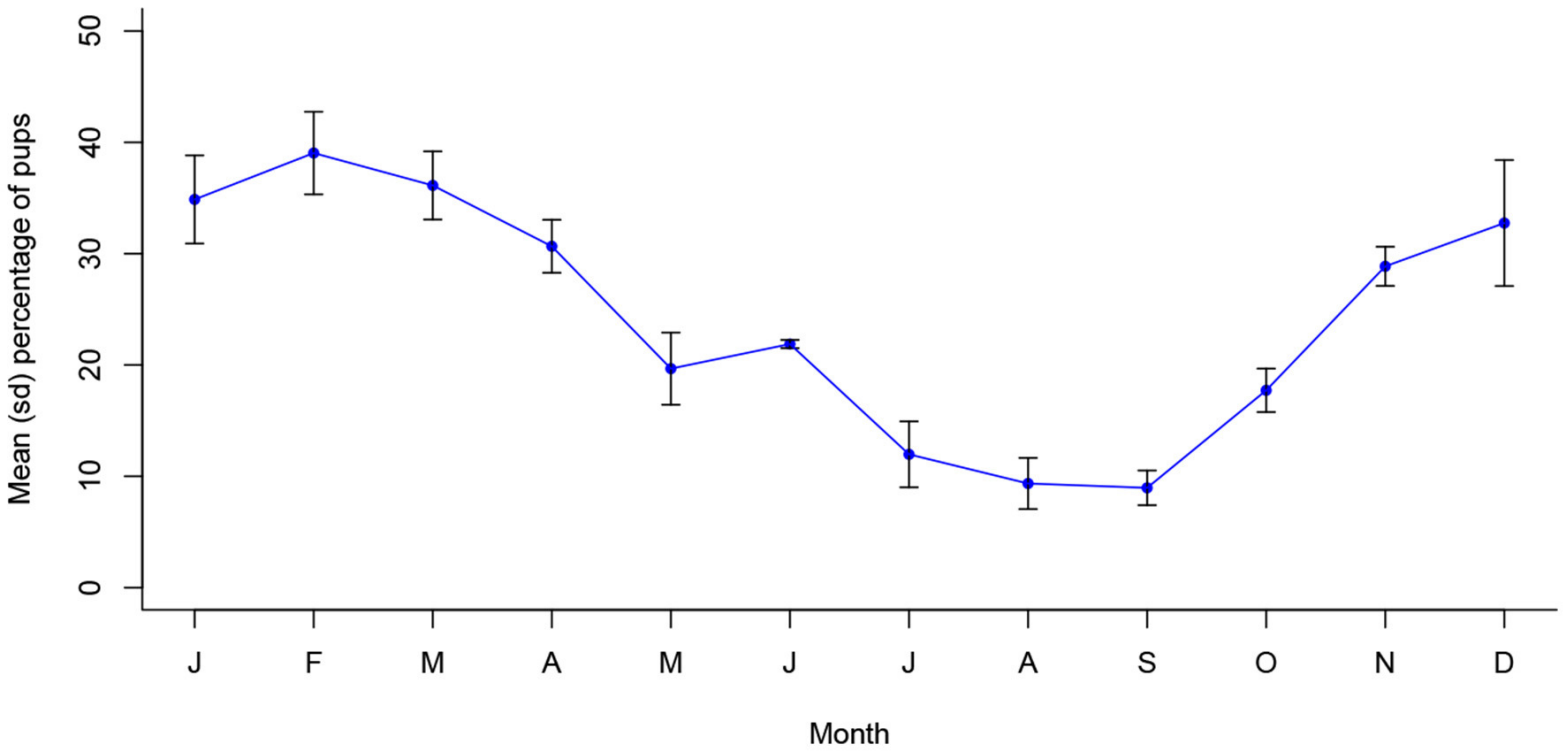
Mean (sd) monthly proportion of pups in Jamshedpur, India between 2013 and 2016 (*n* = 15,610).

Figure 3 depicts annual seasonality of pup proportions in Jamshedpur, which were similarly found to associate significantly with month of the year (*X*^2^ (11, *N* = 15610) = 853.7, *p* < 0.001). Monthly proportions peaked around December to April, following approximately 2 to 3 months after the peak pregnancy period. All three reproductive indicators follow a statistically continuous temporal pattern in Jamshedpur, consisting of a single peak period per year (Appendix A).

### Dehradun

**Figure 4:**
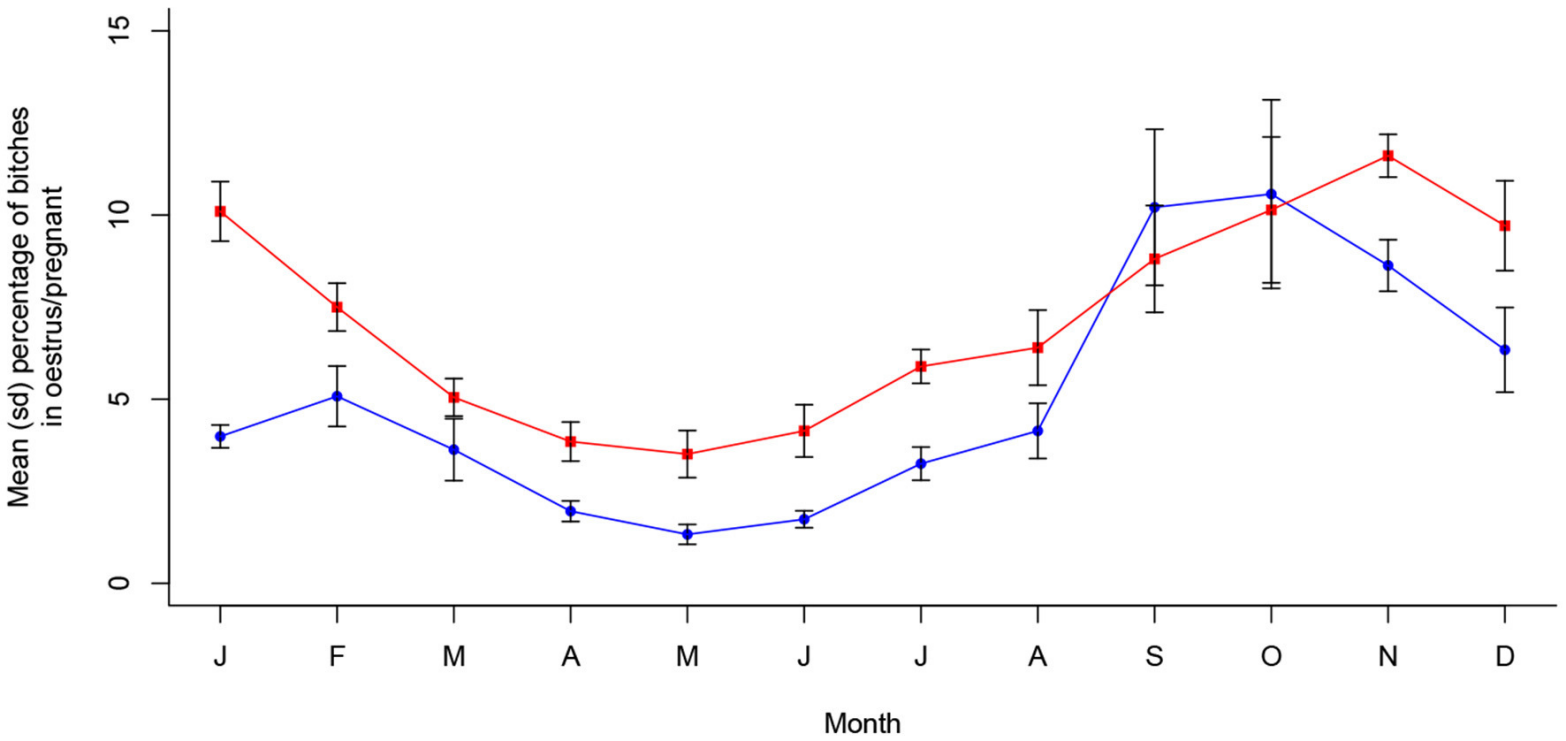
Mean (sd) proportions of bitches in oestrus and pregnant in Dehradun, India between 2016 and 2021 (*n* = 15,565).

Oestrus prevalence was also associated with month of the year in Dehradun (*X*^2^ (11, *N* = 15,565) = 272.89, *p* < 0.001), with a single peak oestrus period occurring from September to November, seeming to reach a maximum approximately a month after that in Jamshedpur. Pregnancy prevalence was also seasonal (*X*^2^ (11, *N* = 15,565) = 149.87, *p* < 0.001), following oestrus period predictably to peak around October through to January. These dynamics are shown in Figure 4 and are statistically temporally consistent with only a single peak period per year (see Appendix A).

**Figure 5:**
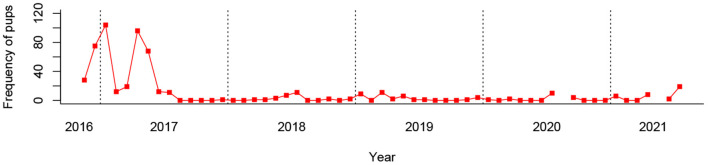
Number of pups sterilized by month in Dehradun, India between 2016 and 2021.

From a mean monthly total of 526.0 dogs, 10.0 pups were sterilized on average per month in Dehradun. However, of the 57 months recorded, over 40% (24) did not record a single pup (see Figure 5), and mean monthly pups falls to only 2.4 when the first 9 months of data recording are excluded. This is the product of pup catching being abandoned early in the programme following an anesthetic mortality. At such low frequencies, clinic pup records are considered to be an unreliable indicator of monthly pup proportions, and thus were not tested for seasonality. It is unclear from our data whether the low proportion of pups entering Dehradun's sterilization clinic is a product of city-specific catching biases or challenges, or a direct result of the city's ongoing sterilization programme.

### Vadodara

In Vadodara, oestrus prevalence was associated with month of the year (*X*^2^ (11, *N* = 8860) = 173.24, *p* < 0.001); however, it is notable that the number of bitches sterilized in oestrus dropped significantly after the first 5 months of sterilization, with only 39 (30%) of the total 126 recorded sterilized after these initial 5 months. It is reasonable to assume that the non-significant month of October, during the otherwise statistically continuous single peak of increased oestrus prevalence during September to December, is simply the result of a very low sample size (see Appendix A); however, it is perhaps more instructive to turn to pregnancy data for a more reliable indication of annual reproductive seasonality.

**Figure 6:**
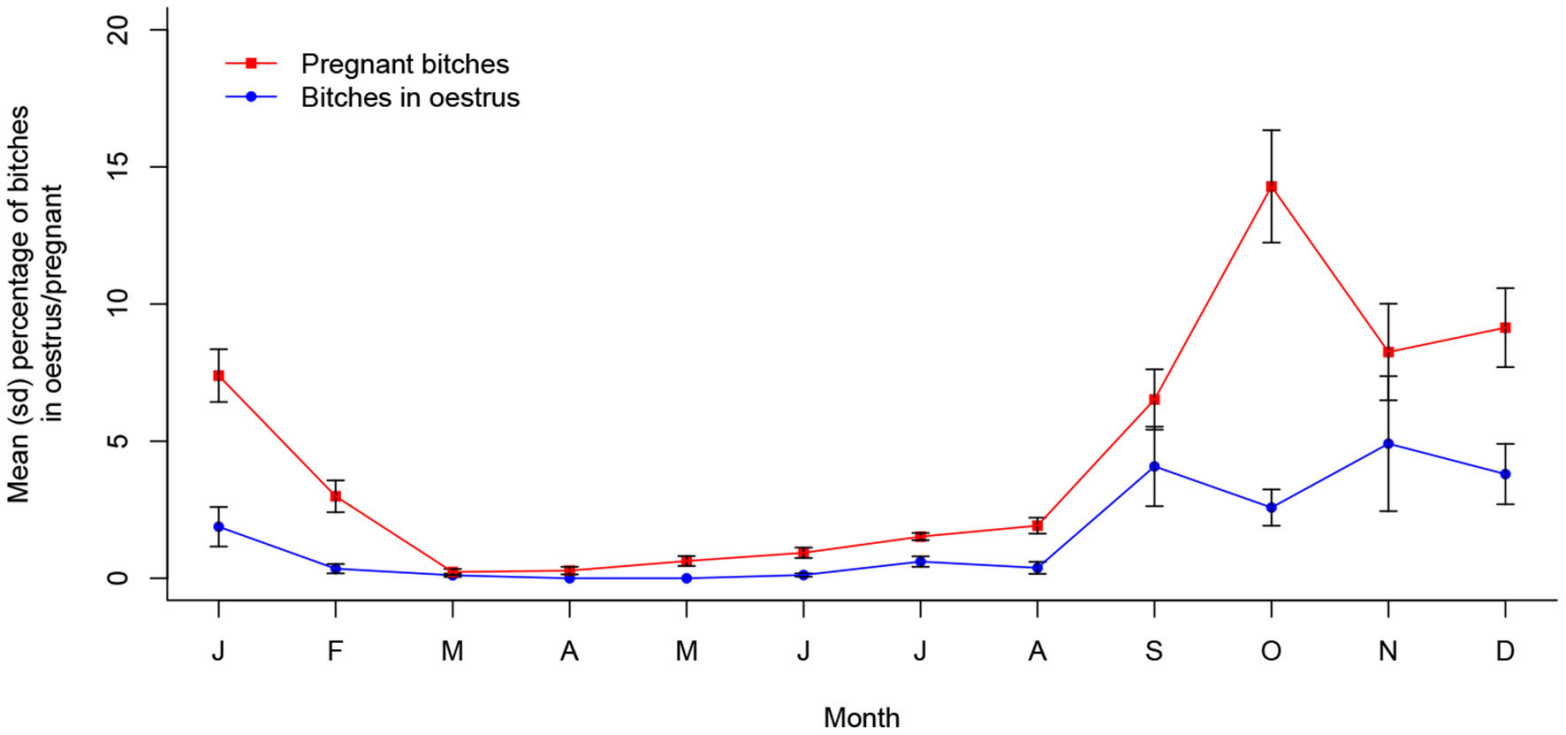
Mean (sd) proportions of bitches in oestrus and pregnant in Vadodara, India between 2017 and 2021 (*n* = 8,860).

**Figure 7:**
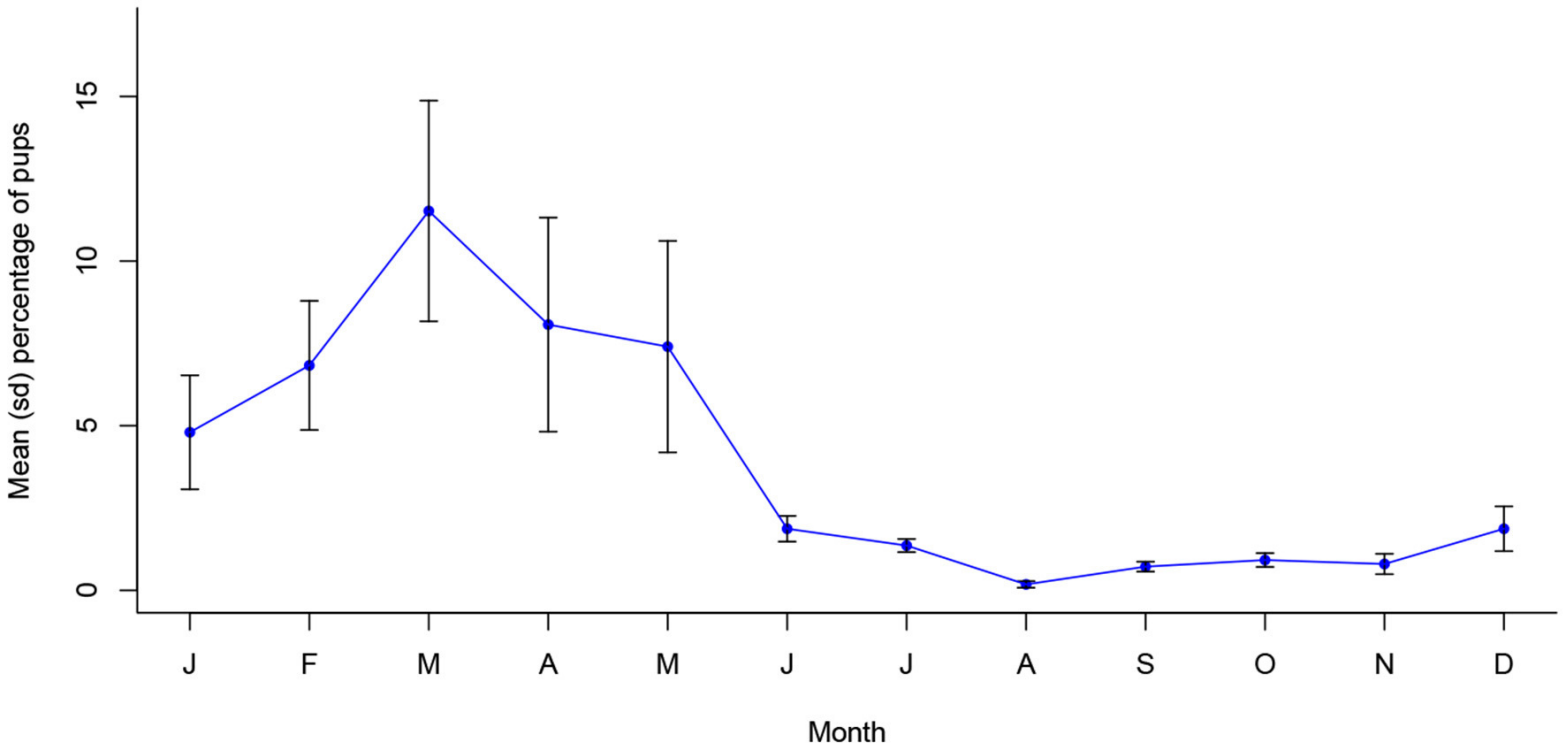
Mean (sd) monthly proportion of pups in Vadodara, India between 2017 and 2021 (*n* = 19,978).

The proportion of pregnant bitches sterilized was significantly associated with month of the year in Vadodara (*X*^2^ (11, *N* = 8,860) = 378.71, *p* < 0.001), with a significantly temporally continuous peak between September and January (see Appendix A). This supports the dynamic that appears to be present in the prevalence of oestrus bitches (Figure 6). Further, pup proportions in Vadodara were associated with month of the year (*X*^2^ (11, *N* = 19,978) = 661.26, *p* < 0.001), following a statistically temporally consistent annual seasonality (see Appendix A), with a single peak in proportions occurring in March (Figure 7).

It is notable that in the case of pups (as for bitches in oestrus), proportions have dropped dramatically over the period of 2017 to 2021: 2018 saw a total of 9.4% (558) pups, compared to 3.8% (156) and 1.0% (47) in 2019 and 2020, respectively. Assuming no major changes have been applied to clinic catching methods, it appears likely that these annual reductions in the prevalence of reproductive indicators are a direct result of the city's sterilization programme.

### Litter Size

Based on fetal counts recorded during HSI clinic abortions of pregnant bitches, the median litter size was six with a standard deviation of 1.90 (*N* = 2,326). Median litter size in Jamshedpur was five (SD = 1.86, *n* = 908); in Dehradun, six (SD = 1.93, *n* = 1,050); and in Vadodara, five (SD = 1.56, *n* = 368). Maximum fetal counts were 18, 13, and 10, respectively.

## Discussion

Our data support the existence of a once-annual seasonal oestrus in free-roaming dog populations of the Indian cities Jamshedpur, Dehradun and Vadodara, with a peak in oestrus and, subsequently, pregnancy occurring during the late/post-monsoon season (August to December). Pup proportions rise soon after, reaching a peak period in winter through early summer, with the highest proportions passing through HSI clinics from February to March. There is a small degree of inter-city variation in seasonality, with the oestrus period (and subsequent peak in pregnancies) in Vadodara and Dehradun lagging approximately a month behind that in Jamshedpur. These post-monsoon reproductive dynamics are consistent with those found in other Indian cities and towns (2–5, 7). Inter-year variation was not tested, however standard deviation bars depicted in each of the figures indicate consistency of the observed seasonality.

### Factors Influencing Breeding Seasonality

In considering the effect of environmental influences on breeding seasonality, it is useful to consider the degree to which such factors may be relevant on the long-term and short-term effect on breeding seasonality. Indeed, in most wild carnivores, breeding seasonality appears to exhibit characteristic seasonal dynamics in temperate and arctic clines, and non-seasonal patterns in equatorial and tropical regions (18). Wild Canids, although also susceptible to variation in dietary and local climatic influences, tend to exhibit consistent seasonal dynamics, captive or otherwise (12, 19, 20). It has been suggested, however, that *C. familiaris* may be considered a non-seasonal breeder, for although breeding may tend toward seasonal dynamics as a result of external influences, the underlying breeding physiology appears non-seasonal, and thus plastic to new or changing environmental conditions to a far greater degree than in other Canid species (Feinstein & Coppinger, 2013). It is speculated that this loss in ‘intrinsic’ seasonality may be the product of relaxed selective pressures in domestic (and especially confined) environments (e.g., non-seasonal food provisioning and reduction of climatic variation), and possibly even artificial selection for increased fecundity (12).

Thus, the question then becomes, what influences are the cause of seasonal breeding tendencies in the street dog populations discussed, and specifically, why post-monsoon? A number of factors have been identified, including increased food availability, artificially altered exercise and housing conditions, favorable climatic conditions, precipitation levels and sensitivity to daylight hours (3, 6, 7, 9).

Photoperiod, specifically day-length, appears to be the most pertinent cue in mammalian reproductive seasonality (17, 18, 21–23), mediated via a melatonin-based regulation pathway initiated at the retina (22, 24). Photoperiod provides a predictable cue to adjust breeding efforts to coincide with peak food availabilities, typically associated with annual rainfall fluctuations in tropical regions (25). While the majority of reproductive seasonality is thought to be controlled by photoperiod, a more basal condition-dependent control—such that reproductive effort is moderated by energy balance—ensures reproductive failure in circumstances of energy deficit (18, 21, 26), whether linked to seasonal fluctuation or otherwise. Additionally, other seasonal cues may act to trigger seasonal reproductive dynamics independent of or in addition to photoperiod, particularly in regions of lesser correlation between food availability and day length, such as arid and equatorial environments (27). Such secondary triggers include rainfall (27), via its impact on pheromone dissipation for example (6), temperature (17, 27), and other more species-specific triggers (26). In the case of the latitudes represented in this study, where little seasonal variation in photoperiod occurs, it may be that such secondary triggers are pertinent.

Concerning domestic dog breeds specifically, many researchers have connected seasonal reproduction to such environmental factors, across a range of latitudes and climatic conditions [e.g. (2, 6, 8, 28, 29)], with others suggesting that a reduced reproductive seasonality in confined domestic dogs is indicative of the climatically buffered and artificially managed conditions of human confinement (9, 30). Even so, it is notable that many domestic dog populations do not exhibit seasonal breeding tendencies (10, 31): and few studies have correlated breeding dynamics to the environmental variables directly, rather testing against months of the year, as in the analysis presented here. However, in two street dog populations of Southern India, (7) showed that environmental conditions—humidity, temperature and especially hours of daylight (photoperiod)—prior to conception are a significant predictor of pregnancy prevalence, indicating a direct correlation between environmental conditions and reproduction as evidenced in other mammals and wild canids (see previous paragraph). Notably, the effect was found to be more distinct in Goa than in Nilgiris, where seasonal climatic conditions are more dramatically delineated; this is perhaps relevant for the consideration of similar local differences between Dehradun, Vadodara and Jamshedpur—cities which are spread widely across India with notable climatic variation.

Another consideration for canids living alongside human populations is human influence. Domestic dogs are heavily dependent on human populations: the extent of which varies from simple food provisioning in the form of human waste and handouts in free-roaming street dogs, to total artificial control of a dog population's diet, lifestyle, breeding and environment (9, 30). With regard to the free-roaming populations in this study, previous HSI household surveys have indicated that, in the majority of Indian cities surveyed, 50–75% of individuals report to feeding street dogs at least once a week (HSI, unpublished data). Dietary quality and caloric intake can significantly affect reproductive capacity, with well provisioned populations sometimes able to achieve oestrus twice per year, while starved populations may omit an oestrus season altogether (12). Therefore, should food availability fluctuate in accordance with seasonal changes in human activity and provisioning, we may assume that this will have an impact on street dog breeding seasonality. This raises an interesting question as to inter-year variation. For example, whether the scarcity of human activity during the Covid-19 pandemic may have influenced breeding seasonality or the magnitude of reproductive indicators during 2020–21. The effects of such should be considered in terms of data collection during this period in Dehradun and Vadodara; the analysis of cross-year averages as were conducted by this study should minimize any confounding effect on the findings.

Finally, we should not dismiss the potential impact of the ongoing street dog management programmes in each of the study cities. Each city has a unique history of evolving population management and ABC efforts, and it must be considered that such interventions, and any seeming seasonal variation within them, may in turn have impacted the seasonal breeding structures of the populations they address. Furthermore, city-specific clinic catching methodologies, urban environments, and circumstances, particularly with regards to changing seasons may also have influenced the data. Such factors are likely to be especially significant in producing the inter-city variation in magnitude (both in terms of mean and peak proportions) of the reproductive indicators measured.

Unfortunately, the count data of each reproductive indicator collected in this study do not provide sample sizes large enough for robust inter-year or inter-city analysis of the precise monthly peaks and shifts in their location by year or city. Furthermore, large variation in monthly catches, including missing months in the case of Vadodara and Dehradun (see Table 1) mean that the precise temporal reproductive fluctuations for single years are difficult to observe with accuracy in this data. This is regrettable, and greater research into precise inter-city seasonality variation and the year-to-year changes is warranted.

### Breeding Seasonality in ABC Programme Design

Understanding the breeding seasonality of a street dog population is essential in order to optimize all stages of population management programmes, from initial monitoring to sterilization efficiency and welfare considerations. In particular, information on breeding seasonality may inform the most efficient means by which to stretch finite and limited resources and funding.

Firstly, with regard to population monitoring, both when establishing a pre-programme baseline and in evaluating the ongoing efficacy of an ABC programme, it is necessary to consider seasonal fluctuations in population abundance the result of annual breeding dynamics. As such, annual or multiannual surveys of street dog abundance must consider the temporal distribution of data collection in order to avoid inherent annual population fluctuations skewing longitudinal survey estimates.

Secondly, breeding seasonality may provide insight as to employing sterilization efforts with maximum efficiency (7). Previous programmes and research simulations show that a female-centric approach to sterilization is most effective in population reduction efforts, as well with regards to reducing negative impacts on welfare (14, 32); knowledge of breeding seasonality may inform on when best to spay the females of the population. It has been suggested that highest impact may be achieved by sterilizing during pregnancy (3), however sterilization prior to peak pregnancy and pup whelping may also achieve high efficacy (2), while additionally addressing population welfare by avoiding the removal of nursing mothers. In either case, it is clear that focusing sterilization efforts prior to peak pup season is likely to be the most efficient approach. Bitches may also be easier to locate and catch prior to the whelping period. Should a programme instead focus on sterilization of pups after birth, not only will more sterilizations be required, but efficiency will drop considerably as few pups born (and subsequently sterilized) survive long enough to breed (4, 33). Furthermore, while early-age neutering appears to be generally safe in dogs (34), there is evidence of (sex- and breed-dependent) health implications of prepubertal sterilization (35–37).

## Conclusion

This study provides further cross-city evidence for a post-monsoon breeding season in Indian free-roaming street dogs: information that is of critical importance in the implementation of cost-effective and welfare-sensitive street dog ABC programmes. This study also supports previous research that the specific seasonal reproductive dynamics of street dog populations are subject to localized variation. It is not fully understood how external factors cause this variation, yet it is clear that an interplay of both climatic and human factors is involved. Further research on the role of such factors as drivers and causes of seasonal breeding variation in the domestic dog is needed in order to refine our understanding of the reproductive landscape onto which we implement street dog ABC programmes. In the meantime, we echo the assertions of other researchers concerning the importance of investigating population specific breeding seasonality in the implementation of such programmes, as well as encouraging in-depth data recording of reproductive and demographic variables by sterilization clinics for the purposes of doing so.

## Data Availability Statement

The original contributions presented in the study are included in the article/Supplementary Material, further inquiries can be directed to the corresponding author/s.

## Ethics Statement

Ethical review and approval was not required for the animal study because the data presented in the manuscript are secondary data from the street dog sterilization program and collected as a part of the standard operating procedure. The animals were not handled for this study purpose, rather we are using an already existing dataset to find the research results.

## Author Contributions

Conceptualization: GB, TK, and AC. Data collection: DY, MB, CP, SS, PP, BP, and AC. Software, data analysis and visualization, and writing—original draft: GB. Writing—review & editing: AC, TK, DY, MB, CP, SS, PP, GB, and BP. All authors contributed to the article and approved the submitted version.

## Conflict of Interest

The authors declare that the research was conducted in the absence of any commercial or financial relationships that could be construed as a potential conflict of interest.

## Publisher's Note

All claims expressed in this article are solely those of the authors and do not necessarily represent those of their affiliated organizations, or those of the publisher, the editors and the reviewers. Any product that may be evaluated in this article, or claim that may be made by its manufacturer, is not guaranteed or endorsed by the publisher.
