# Supplementary material for: Seasonal Patterns of Oestrus and Reproduction in Street Dogs of Indian Cities
Source: Front Vet Sci. 2022 Jun 17;9:821424. doi: 10.3389/fvets.2022.821424 (PMC9247615; doi:10.3389/fvets.2022.821424)
Supplement: Supplementary file 1 [file Table_1.DOCX]

Seasonal Patterns of Oestrus and Reproduction in Street Dogs of Indian Cities

# Appendix A: Statistical analyses

## TABLE A1. Prevalence of reproductive indicators (bitches in oestrus, pregnant bitches, and pups) in Jamshedpur, India (2013-2016). Post-hoc chi-squared tests reported as: Residuals (p-value); *significantly lesser proportion, ºsignificantly greater proportion

| Month | Oestrus (counts do not include pups) | | | Pregnancy (counts do not include pups) | | | Pups | | |
| --- | --- | --- | --- | --- | --- | --- | --- | --- | --- |
|  | Bitches not in oestrus | Bitches in oestrus | Chi-squared post-hoc (Bonferroni) | Bitches not pregnant | Pregnant bitches | Chi-squared post-hoc  (Bonferroni) | Adult dogs | Pups | Chi-squared  post-hoc  (Bonferroni) |
| Jan | 559 | 21 | -3.940 (0.002)* | 471 | 109 | 2.091 (0.877) | 1178 | 618 | 8.569 (<0.001)º |
| Feb | 572 | 10 | -5.755 (<0.001)* | 499 | 83 | -1.068 (1.000) | 1109 | 701 | 13.059 (<0.001)º |
| Mar | 541 | 11 | -5.335 (<0.001)* | 509 | 43 | -5.422 (<0.001)* | 1085 | 610 | 9.862 (<0.001)º |
| Apr | 379 | 9 | -4.155 (<0.001)* | 373 | 15 | -6.671 (<0.001)* | 790 | 337 | 3.051 (0.055) |
| May | 239 | 8 | -2.721 (0.156) | 233 | 14 | -4.460 (<0.001)* | 526 | 130 | -3.722 (0.005)* |
| Jun | 412 | 36 | 0.216 (1.000) | 422 | 26 | -6.037 (<0.001)* | 886 | 247 | -3.392 (0.017)* |
| Jul | 483 | 54 | 2.074 (0.914) | 471 | 66 | -2.336 (0.467) | 1054 | 143 | -11.577 (<0.001)* |
| Aug | 429 | 97 | 9.586 (<0.001)º | 445 | 81 | -0.259 (1.000) | 1038 | 109 | -13.271 (<0.001)* |
| Sep | 381 | 68 | 6.075 (<0.001)º | 299 | 150 | 10.661 (<0.001)º | 887 | 86 | -12.638 (<0.001)* |
| Oct | 421 | 51 | 2.567 (0.246) | 344 | 128 | 7.044 (<0.001)º | 968 | 204 | -7.018 (<0.001)* |
| Nov | 439 | 45 | 1.308 (1.000) | 383 | 101 | 3.200 (0.033)º | 1000 | 389 | 1.731 (1.000) |
| Dec | 448 | 37 | -0.125 (1.000) | 393 | 92 | 2.006 (1.000) | 1021 | 494 | 6.109 (<0.001)º |
| Chi-squared | X-squared = 221.72, df = 11, p-value < 0.001 | | | X-squared = 294.07, df = 11, p-value < 0.001 | | | X-squared = 853.7, df = 11, p-value < 0.001 | | |

## TABLE A2. Prevalence of reproductive indicators (bitches in oestrus, pregnant bitches) in Dehradun, India (2016-2021). Post-hoc chi-squared tests reported as: Residuals (p-value); *significantly lesser proportion, ºsignificantly greater proportion

| Month | Oestrus (counts do not include pups) | | | Pregnancy (counts do not include pups) | | |
| --- | --- | --- | --- | --- | --- | --- |
|  | Bitches not in oestrus | Bitches in oestrus | Chi-squared post-hoc (Bonferroni) | Bitches not pregnant | Pregnant bitches | Chi-squared post-hoc  (Bonferroni) |
| Jan | 1226 | 51 | -0.961 (1.000) | 1148 | 129 | 4.990 (<0.001)º |
| Feb | 1569 | 84 | 1.142 (1.000) | 1529 | 124 | 1.296 (1.000) |
| Mar | 1565 | 59 | -1.836 (1.000) | 1542 | 82 | -2.880 (0.095) |
| Apr | 1399 | 28 | -4.893 (<0.001)* | 1372 | 55 | -4.570 (<0.001)* |
| May | 1266 | 17 | -5.762 (<0.001)* | 1238 | 45 | -4.828 (<0.001)* |
| Jun | 1354 | 24 | -5.212 (<0.001)* | 1321 | 57 | -4.045 (0.001)* |
| Jul | 1724 | 58 | -2.750 (0.143) | 1677 | 105 | -1.527 (1.000) |
| Aug | 1482 | 64 | -0.776 (1.000) | 1447 | 99 | -0.565 (1.000) |
| Sep | 1090 | 124 | 9.920 (<0.001)º | 1107 | 107 | 2.992 (0.067) |
| Oct | 626 | 74 | 7.866 (<0.001)º | 629 | 71 | 3.666 (0.006)º |
| Nov | 614 | 58 | 5.227 (<0.001)º | 594 | 78 | 5.136 (<0.001)º |
| Dec | 945 | 64 | 2.865 (0.100) | 911 | 98 | 3.885 (0.002)º |
| Chi-squared | X-squared = 272.89, df = 11, p-value < 0.001 | | | X-squared = 149.87, df = 11, p-value < 0.001 | | |

## TABLE A3. Prevalence of reproductive indicators (bitches in oestrus, pregnant bitches, and pups) in Vadodara, India (2017-2021). Post-hoc chi-squared tests reported as: Residuals (p-value); *significantly lesser proportion, ºsignificantly greater proportion

| Month | Oestrus (counts do not include pups) | | | Pregnancy (counts do not include pups) | | | Pups | | |
| --- | --- | --- | --- | --- | --- | --- | --- | --- | --- |
|  | Bitches not in oestrus | Bitches in oestrus | Chi-squared post-hoc (Bonferroni) | Bitches not pregnant | Pregnant bitches | Chi-squared post-hoc  (Bonferroni) | Adult dogs | Pups | Chi-squared  post-hoc  (Bonferroni) |
| Jan | 836 | 16 | 1.182 (1.000) | 789 | 63 | 4.987 (<0.001)º | 1965 | 99 | 1.673 (1.000) |
| Feb | 866 | 3 | -2.823 (0.114) | 843 | 26 | -1.807 (1.000) | 1922 | 141 | 6.601 (<0.001)º |
| Mar | 870 | 1 | -3.432 (0.014)* | 869 | 2 | -6.112 (<0.001)* | 1752 | 228 | 17.511 (<0.001)º |
| Apr | 725 | 0 | -3.375 (0.018)* | 723 | 2 | -5.461 (<0.001)* | 1413 | 124 | 8.151 (<0.001)º |
| May | 640 | 0 | -3.155 (0.039)* | 636 | 4 | -4.645 (<0.001)* | 1202 | 96 | 6.181 (<0.001)º |
| Jun | 855 | 1 | -3.394 (0.017)* | 848 | 8 | -4.966 (<0.001)* | 1679 | 32 | -4.872 (<0.001)* |
| Jul | 982 | 6 | -2.295 (0.522) | 973 | 15 | -4.04 (<0.001)* | 1811 | 25 | -6.216 (<0.001)* |
| Aug | 520 | 2 | -2.067 (0.930) | 512 | 10 | -2.641 (0.198) | 1128 | 2 | -6.851 (<0.001)* |
| Sep | 706 | 30 | 6.350 (<0.001)º | 688 | 48 | 3.363 (0.019)º | 1518 | 11 | -6.943 (<0.001)* |
| Oct | 566 | 15 | 2.442 (0.350) | 498 | 83 | 12.663 (<0.001)º | 1292 | 12 | -5.995 (<0.001)* |
| Nov | 484 | 25 | 6.849 (<0.001)º | 467 | 42 | 4.773 (<0.001)º | 1487 | 12 | -6.704 (<0.001)* |
| Dec | 684 | 27 | 5.578 (<0.001)º | 646 | 65 | 6.952 (<0.001)º | 1989 | 38 | -5.338 (<0.001)* |
| Chi-squared | X-squared = 173.24, df = 11, p-value < 0.001 | | | X-squared = 378.71, df = 11, p-value < 0.001 | | | X-squared = 661.26, df = 11, p-value < 0.001 | | |

# Appendix B: Tables of data

TABLE B1. Sterilisation records by month in Jamshedpur, India (2013-2016)

| Month | Total dogs | Total bitches (exc. pups) | Bitches in oestrus | Bitches pregnant | Pups | Number of foetuses |
| --- | --- | --- | --- | --- | --- | --- |
| 2013 |  |  |  |  |  |  |
| Jul | 98 | 49 | 4 | 1 | 7 | 8 |
| Aug | 61 | 26 | 2 | 6 | 5 | 33 |
| Sep | 132 | 64 | 23 | 11 | 14 | 70 |
| Oct | 286 | 104 | 16 | 24 | 45 | 129 |
| Nov | 411 | 148 | 10 | 26 | 115 | 140 |
| Dec | 458 | 158 | 17 | 27 | 115 | 136 |
| 2014 |  |  |  |  |  |  |
| Jan | 563 | 218 | 15 | 43 | 140 | 227 |
| Feb | 558 | 196 | 2 | 38 | 166 | 194 |
| Mar | 547 | 208 | 5 | 18 | 168 | 91 |
| Apr | 577 | 181 | 4 | 13 | 182 | 64 |
| May | 389 | 139 | 4 | 10 | 81 | 53 |
| Jun | 587 | 233 | 23 | 15 | 119 | 84 |
| Jul | 600 | 262 | 31 | 45 | 76 | 258 |
| Aug | 561 | 251 | 57 | 48 | 57 | 291 |
| Sep | 413 | 194 | 25 | 85 | 45 | 457 |
| Oct | 415 | 182 | 19 | 58 | 66 | 326 |
| Nov | 571 | 212 | 22 | 40 | 153 | 223 |
| Dec | 450 | 170 | 4 | 37 | 111 | 168 |
| 2015 |  |  |  |  |  |  |
| Jan | 622 | 208 | 2 | 42 | 196 | 216 |
| Feb | 599 | 205 | 6 | 27 | 226 | 152 |
| Mar | 561 | 184 | 5 | 10 | 176 | 39 |
| Apr | 550 | 207 | 5 | 2 | 155 | 9 |
| May | 267 | 108 | 4 | 4 | 49 | 24 |
| Jun | 546 | 215 | 13 | 11 | 128 | 60 |
| Jul | 499 | 226 | 19 | 20 | 60 | 101 |
| Aug | 525 | 249 | 38 | 27 | 47 | 162 |
| Sep | 428 | 191 | 20 | 54 | 27 | 308 |
| Oct | 471 | 186 | 16 | 46 | 93 | 248 |
| Nov | 407 | 124 | 13 | 35 | 121 | 185 |
| Dec | 607 | 157 | 16 | 28 | 268 | 133 |
| 2016 |  |  |  |  |  |  |
| Jan | 611 | 154 | 4 | 24 | 282 | 109 |
| Feb | 653 | 181 | 2 | 18 | 309 | 96 |
| Mar | 587 | 160 | 1 | 15 | 266 | 71 |
|  |  |  |  |  |  |  |
| Total | 15610 | 5750 | 447 | 908 | 4068 | 4865 |

TABLE B2. Sterilisation records by month in Dehradun, India (2016-2021)

| Month | Total dogs | Total bitches (exc. pups) | Bitches in oestrus | Bitches pregnant | Pups | Number of foetuses |
| --- | --- | --- | --- | --- | --- | --- |
| 2016 |  |  |  |  |  |  |
| Nov | 165 | 58 | 4 | 12 | 28 | 80 |
| Dec | 610 | 275 | 30 | 38 | 75 | 250 |
| 2017 |  |  |  |  |  |  |
| Jan | 754 | 347 | 14 | 41 | 104 | 259 |
| Feb | 791 | 420 | 10 | 36 | 12 | 219 |
| Mar | 733 | 371 | 3 | 16 | 19 | 108 |
| Apr | 829 | 414 | 7 | 17 | 96 | 98 |
| May | 843 | 420 | 6 | 19 | 98 | 99 |
| Jun | 840 | 442 | 5 | 26 | 12 | 161 |
| Jul | 870 | 447 | 7 | 32 | 11 | 233 |
| Aug | 1007 | 448 | 11 | 18 | 0 | 134 |
| Sep | 1059 | 476 | 31 | 41 | 0 | 272 |
| Oct | 368 | 179 | 16 | 18 | 0 | 106 |
| Nov | 396 | 193 | 16 | 15 | 0 | 98 |
| Dec | 426 | 168 | 3 | 8 | 1 | 58 |
| 2018 |  |  |  |  |  |  |
| Jan | 535 | 227 | 8 | 31 | 0 | 178 |
| Feb | 502 | 255 | 12 | 18 | 0 | 106 |
| Mar | 670 | 376 | 16 | 21 | 1 | 124 |
| Apr | 766 | 524 | 9 | 19 | 1 | 127 |
| May | 790 | 508 | 2 | 16 | 3 | 111 |
| Jun | 492 | 308 | 3 | 14 | 7 | 92 |
| Jul | 572 | 364 | 4 | 12 | 11 | 92 |
| Aug | 802 | 545 | 13 | 46 | 0 | 289 |
| Sep | 548 | 283 | 18 | 24 | 0 | 163 |
| Oct | 373 | 150 | 15 | 11 | 2 | 78 |
| Nov | 270 | 147 | 15 | 16 | 0 | 84 |
| Dec | 473 | 219 | 11 | 25 | 2 | 139 |
| 2019 |  |  |  |  |  |  |
| Jan | 528 | 210 | 7 | 15 | 9 | 92 |
| Feb | 547 | 251 | 11 | 18 | 0 | 106 |
| Mar | 644 | 294 | 4 | 13 | 11 | 78 |
| Apr | 617 | 304 | 3 | 11 | 2 | 60 |
| May | 543 | 309 | 8 | 7 | 6 | 41 |
| Jun | 557 | 306 | 10 | 11 | 1 | 61 |
| Jul | 598 | 330 | 12 | 14 | 1 | 75 |
| Aug | 404 | 217 | 7 | 9 | 0 | 49 |
| Sep | 316 | 166 | 8 | 3 | 0 | 21 |
| Oct | 171 | 106 | 0 | 5 | 0 | 29 |
| Nov | 283 | 138 | 12 | 13 | 1 | 63 |
| Dec | 578 | 250 | 2 | 17 | 4 | 89 |
| 2020 |  |  |  |  |  |  |
| Jan | 507 | 228 | 7 | 20 | 1 | 117 |
| Feb | 576 | 291 | 10 | 15 | 0 | 97 |
| Mar | 264 | 123 | 2 | 5 | 2 | 18 |
| Apr | 0 | 0 | 0 | 0 | 0 | 0 |
| May | 73 | 46 | 1 | 3 | 0 | 15 |
| Jun | 518 | 296 | 4 | 6 | 0 | 40 |
| Jul | 591 | 284 | 10 | 23 | 10 | 135 |
| Aug | 580 | 336 | 33 | 26 | 0 | 166 |
| Sep | 387 | 289 | 67 | 39 | 4 | 250 |
| Oct | 483 | 265 | 43 | 37 | 0 | 224 |
| Nov | 285 | 136 | 11 | 22 | 0 | 125 |
| Dec | 151 | 97 | 18 | 10 | 0 | 54 |
| 2021 |  |  |  |  |  |  |
| Jan | 575 | 265 | 15 | 22 | 6 | 134 |
| Feb | 804 | 436 | 41 | 37 | 0 | 207 |
| Mar | 874 | 460 | 34 | 27 | 0 | 165 |
| Apr | 385 | 185 | 9 | 8 | 8 | 49 |
| May | 0 | 0 | 0 | 0 | 0 | 0 |
| Jun | 39 | 26 | 2 | 0 | 2 | 0 |
| Jul | 620 | 357 | 25 | 24 | 19 | 148 |
|  |  |  |  |  |  |  |
| Total | 29982 | 15565 | 705 | 1050 | 570 | 6466 |

TABLE B3. Sterilisation records by month in Vadodara, India (2017-2021)

| Month | Total dogs | | Total bitches (exc. pups) | Bitches in oestrus | Bitches pregnant | Pups | Number of foetuses |
| --- | --- | --- | --- | --- | --- | --- | --- |
| 2017 |  | |  |  |  |  |  |
| Sep | 253 | | 122 | 23 | 11 | 5 | 61 |
| Oct | 154 | | 67 | 8 | 9 | 2 | 41 |
| Nov | 696 | | 217 | 25 | 23 | 10 | 123 |
| Dec | 702 | | 228 | 18 | 29 | 30 | 134 |
| 2018 |  | |  |  |  |  |  |
| Jan | 703 | | 257 | 13 | 28 | 78 | 135 |
| Feb | 743 | | 305 | 3 | 14 | 94 | 68 |
| Mar | 644 | | 249 | 0 | 0 | 143 | 0 |
| Apr | 678 | | 315 | 0 | 0 | 108 | 0 |
| May | 690 | | 334 | 0 | 2 | 87 | 14 |
| Jun | 502 | | 257 | 0 | 4 | 20 | 29 |
| Jul | 452 | | 222 | 0 | 4 | 11 | 23 |
| Aug | 487 | | 229 | 0 | 5 | 2 | 26 |
| Sep | 405 | | 202 | 1 | 17 | 4 | 89 |
| Oct | 275 | | 129 | 6 | 24 | 7 | 136 |
| Nov | 152 | | 50 | 0 | 2 | 0 | 11 |
| Dec | 358 | | 136 | 4 | 8 | 4 | 57 |
| 2019 |  | |  |  |  |  |  |
| Jan | 399 | | 176 | 3 | 11 | 2 | 46 |
| Feb | 413 | | 167 | 0 | 3 | 32 | 18 |
| Mar | 475 | | 197 | 1 | 0 | 83 | 0 |
| Apr | 503 | | 242 | 0 | 2 | 16 | 9 |
| May | 399 | | 205 | 0 | 2 | 8 | 13 |
| Jun | 397 | | 191 | 0 | 0 | 5 | 0 |
| Jul | 404 | | 229 | 2 | 5 | 6 | 25 |
| Aug | 262 | | 90 | 0 | 3 | 0 | 13 |
| Sep | 231 | | 107 | 0 | 1 | 0 | 7 |
| Oct | 227 | | 97 | 0 | 14 | 2 | 71 |
| Nov | 230 | | 93 | 0 | 10 | 1 | 47 |
| Dec | 332 | | 118 | 0 | 8 | 1 | 34 |
| 2020 |  | |  |  |  |  |  |
| Jan | 365 | | 166 | 0 | 13 | 9 | 56 |
| Feb | 443 | | 189 | 0 | 4 | 15 | 13 |
| Mar | 202 | | 101 | 0 | 0 | 2 | 0 |
| Apr | 0 | | 0 | 0 | 0 | 0 | 0 |
| May | 116 | | 48 | 0 | 0 | 1 | 0 |
| Jun | 437 | | 218 | 1 | 2 | 7 | 10 |
| Jul | 462 | | 260 | 4 | 4 | 6 | 26 |
| Aug | 381 | | 203 | 2 | 2 | 0 | 13 |
| Sep | 640 | | 305 | 6 | 19 | 2 | 104 |
| Oct | 648 | | 288 | 1 | 36 | 1 | 190 |
| Nov | 421 | | 149 | 0 | 7 | 1 | 38 |
| Dec | 635 | | 229 | 5 | 20 | 3 | 96 |
| 2021 |  |  | |  |  |  |  |
| Jan | 597 | | 253 | 0 | 11 | 10 | 56 |
| Feb | 464 | | 208 | 0 | 5 | 3 | 25 |
| Mar | 659 | | 324 | 0 | 2 | 11 | 12 |
| Apr | 356 | | 168 | 0 | 0 | 11 | 0 |
| May | 93 | | 53 | 0 | 0 | 3 | 0 |
| Jun | 375 | | 190 | 0 | 2 | 10 | 16 |
| Jul | 518 | | 277 | 0 | 2 | 2 | 14 |
|  |  | |  |  |  |  |  |
| Total | 19978 | | 8860 | 126 | 368 | 858 | 1899 |
